# Past and estimated future impact of invasive alien mammals on insular threatened vertebrate populations

**DOI:** 10.1038/ncomms12488

**Published:** 2016-08-18

**Authors:** Erin E. McCreless, David D. Huff, Donald A. Croll, Bernie R. Tershy, Dena R. Spatz, Nick D. Holmes, Stuart H. M. Butchart, Chris Wilcox

**Affiliations:** 1 Department of Ecology and Evolutionary Biology, Long Marine Laboratory, University of California Santa Cruz, 100 Shaffer Road, Santa Cruz, California 95060, USA; 2 Point Adams Research Station, Fish Ecology Division, Northwest Fisheries Science Center, NOAA Fisheries, PO Box 155, Hammond, Oregon 97121, USA; 3 Island Conservation, 2161 Delaware Avenue, Suite A, Santa Cruz, California 95060, USA; 4 BirdLife International, David Attenborough Building, Pembroke Street, Cambridge CB23QZ, UK; 5 Department of Zoology, University of Cambridge, Downing Street, Cambridge CB23EJ, UK; 6 Marine and Atmospheric Research, Commonwealth Scientific and Industrial Research Organization, Hobart, Tasmania 7000, Australia

## Abstract

Invasive mammals on islands pose severe, ongoing threats to global biodiversity. However, the severity of threats from different mammals, and the role of interacting biotic and abiotic factors in driving extinctions, remain poorly understood at a global scale. Here we model global extirpation patterns for island populations of threatened and extinct vertebrates. Extirpations are driven by interacting factors including invasive rats, cats, pigs, mustelids and mongooses, native species taxonomic class and volancy, island size, precipitation and human presence. We show that controlling or eradicating the relevant invasive mammals could prevent 41–75% of predicted future extirpations. The magnitude of benefits varies across species and environments; for example, managing invasive mammals on small, dry islands could halve the extirpation risk for highly threatened birds and mammals, while doing so on large, wet islands may have little benefit. Our results provide quantitative estimates of conservation benefits and, when combined with costs in a return-on-investment framework, can guide efficient conservation strategies.

## Abstract (web summary)

Invasive vertebrates can decimate native species living on islands. Using a model of global extirpation patterns, McCreless *et al*. identify the types of invasive species most harmful to natives and predict when controlling or eradicating the invasive species is likely to succeed as a conservation strategy.

## Graphical abstract

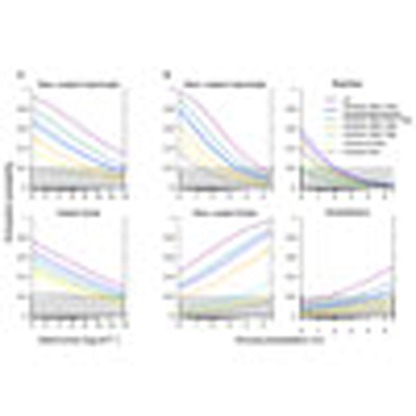

The introduction and spread of invasive alien species (hereafter invasive species) is a leading cause of biodiversity loss^1^. Invasive species are particularly destructive to island species and ecosystems: 75% of recorded terrestrial vertebrate extinctions took place on islands^2^ and most were caused fully or in part by invasive species^3^. Currently, 40% of species threatened with global extinction are island residents^2^. Most archipelagos worldwide have been colonized by invasive species with invasive mammals the most widespread and damaging^4^,^5^.

The degree to which invasive mammals affect insular native species depends on interacting factors including: (1) life history traits of the native species (for example, body size, diet)^6^; (2) life history traits of the invasive mammal; (3) the invasive mammal's ecological interaction with each native species (for example, predation, competition)^7^; and (4) island geologic origin, size, topography and climate^8^. Previous syntheses of impacts have qualitatively characterized threats from particular invasive mammals to island taxa globally^4^,^5^,^7^, or quantified these threats for subsets of islands and taxa^9^,^10^. Understanding and quantifying such effects and their interactions more comprehensively is fundamental to developing robust conservation plans. Efforts to prioritize islands for invasive mammal control and eradication (hereafter invasive management) traditionally depend on expert judgment and ranking systems to predict conservation benefits^11^. These approaches can be based on unquantified assumptions about the effects of invasive mammals and the benefits of managing them, and are likely biased by conventional wisdom and past experience. Quantifying invasive mammal impacts strengthens conservation plans by identifying contexts in which conventional wisdom fails^12^, thus illuminating undetected opportunities and potential failures.

Here we quantify the factors driving island population extirpations globally using the Threatened Island Biodiversity Database^13^—a publically available, comprehensive data set containing current and historic island distributions for highly threatened and extinct terrestrial vertebrates (global International Union for the Conservation of Nature (IUCN) Red List categories of Endangered, Critically Endangered, Extinct in the Wild and Extinct). Our study has three overarching goals: (1) quantify the degree of threat from specific invasive mammals to each native threatened taxon under various environmental conditions, (2) estimate population- and species-level responses of threatened insular species to invasive mammal management and (3) identify contexts in which conventional wisdom on the outcome of managing invasive mammals is not empirically supported.

To achieve our first goal, we generate a set of hypotheses for how different types of invasive mammals, island characteristics and interactions between these biotic and abiotic factors influence extirpation risk for native threatened vertebrate populations globally (Tables 1 and 2). We define extirpation as the disappearance of a species from a single island after AD 1500 (the earliest date for species assessments by the IUCN^14^). We test these hypotheses by fitting a regression model that identifies the invasive mammals, island characteristics and interactive effects most strongly associated with population extirpations on islands. We show that most of the variation in extirpations is explained by a model that includes a suite of interacting biotic and abiotic factors, including the presence of invasive rats, cats, pigs, mustelids and mongooses, native species taxonomic class and flight ability, island size, annual precipitation and human presence.

To achieve our second and third goals, we use the model to estimate population extirpation risk for extant populations of threatened species under current conditions, as well as under potential invasive mammal management scenarios. We estimate that without additional conservation interventions, up to 45% of these populations will be extirpated, but that managing the relevant invasive mammals could prevent 41–75% of predicted extirpations. We highlight contexts in which managing invasive mammals would reduce extinction risk for native species by more than half, as well as those in which interactions between biotic and abiotic factors could make invasive mammal management a less effective conservation strategy.

## Results

### Native and non-native island species distributions

We identified 1,257 highly threatened or historically extinct terrestrial vertebrate species (global IUCN Red List categories of Endangered, Critically Endangered, Extinct in the Wild or Extinct^14^) with 2,656 breeding populations on 1,024 islands worldwide (Supplementary Table 1). Twenty-five percent (651) of these populations were extirpated, with extirpation defined as the disappearance of a species from a single island after AD 1500 (the earliest date for species assessments by the IUCN). For most species that occurred on a single island, extirpation equated to global extinction; however, 31 species (4% of single-island species) also had extant continental populations. We classified the ∼160 documented invasive mammal species into 12 broad groups (Supplementary Table 2); 73% of the islands (747) contained at least one of these invasive mammal groups.

### Model selection and validation

We constructed generalized estimating equations (GEEs)^15^ to build logistic models in which the response variable was the extirpation or persistence of each island population of a threatened or extinct vertebrate species. Native threatened species were classified into six groups: volant birds, non-volant birds, bats (that is, volant mammals), non-volant mammals, amphibians and reptiles (henceforth termed ‘class/volancy'; see Methods and Supplementary Methods). We tested a range of biotic and abiotic covariates (Tables 1 and 2 and Supplementary Data 1) and used QICu^15^ and model error estimates^16^ to choose a final model (Supplementary Fig. 1). The final chosen model contained eight main effects (class/volancy, island area, annual precipitation, human presence and invasive cats, rats, pigs, and mustelids/mongooses (grouped together; see Supplementary Methods)) and five interaction terms (class/volancy*area, class/volancy*precipitation, class/volancy*cat, class/volancy*pig, and rat*area; Supplementary Tables 3 and 4). The area under the receiver operating characteristic curve for the full data set was 0.75 and the mean area under the curve (AUC) across 10,000 K-fold validation runs was 0.70 (s.d. 0.091), indicating that the model was able to discriminate between population persistence and extirpation^17^.

### Effects of area and precipitation on extirpation risk

**Figure 1:**
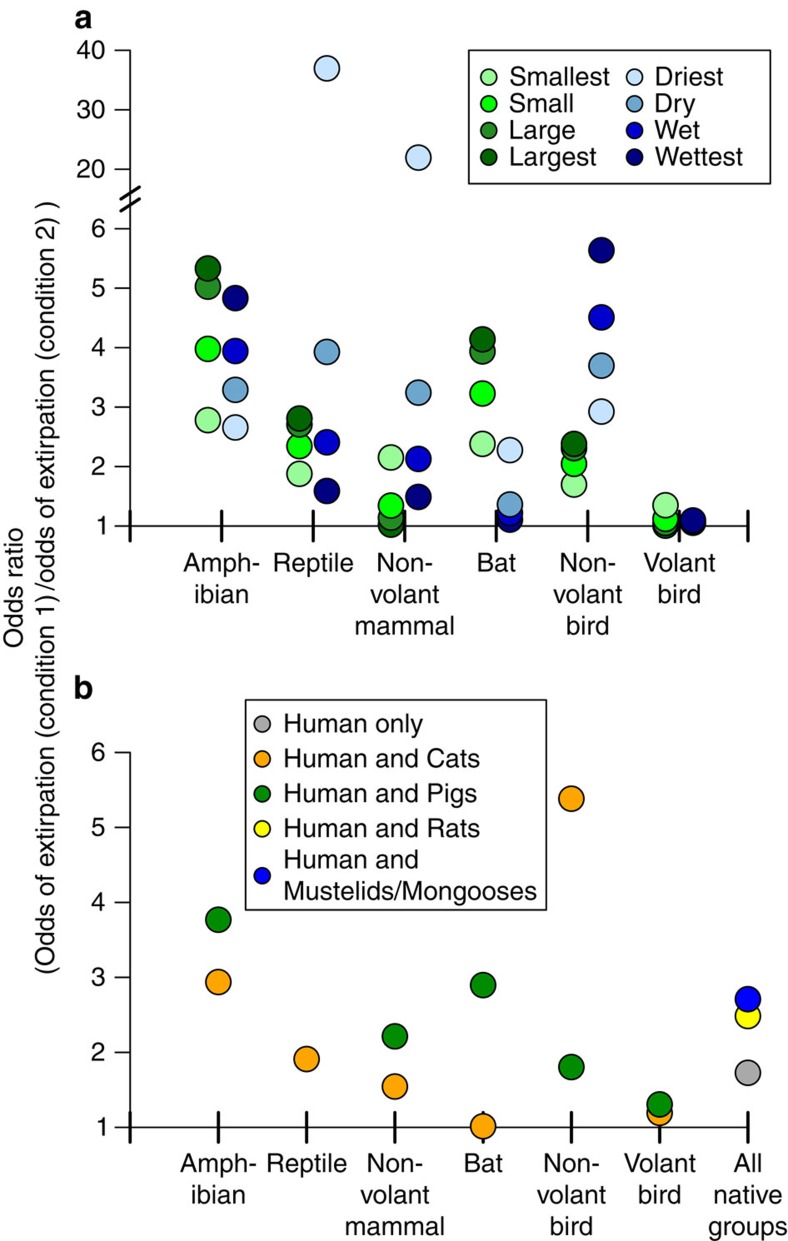
Model predicted odds ratios comparing extirpation risk across island conditions. (**a**) Effects of island area and precipitation. Green: the ratios of the odds of extirpation on the smallest (1.0e^−5^ km^2^) or largest (783,400 km^2^) islands versus the odds on small (first quartile (qu.) area (A): 0.6 km^2^), median (A: 9.7 km^2^), or large (third qu. A: 193.4 km^2^) islands (precipitation held at median value (1,326 mm)). Blue: ratios of the odds of extirpation on the wettest (maximum precipitation (*P*): 5,441 mm) or driest (minimum *P*: 0 mm) islands versus the odds on wet (third qu. *P*: 2,062 mm), median (*P*: 1,326 mm) or dry (first qu. *P*: 702 mm) islands (area held at median value (9.7 km^2^)). (**b**) Effects of human populations and invasive mammals. Grey: the ratio of the odds of extirpation on invasive mammal-free islands with versus without human populations. Yellow and blue: the ratios of the odds of extirpation on inhabited islands containing rats (yellow) or mustelids/mongooses (blue) versus the odds on uninhabited, invasive mammal-free islands, for all native species groups. Orange and green: the ratios of the odds of extirpation on inhabited islands containing invasive cats (orange) or pigs (green) versus the odds on uninhabited, invasive mammal-free islands. The odds ratio for the effect of pigs on native reptile populations is not shown because it is <1 and thus difficult to interpret^64^. Odds ratios in (**a**) were calculated with invasive mammals absent, and in (**b**) with area and precipitation held constant at the median values for inhabited islands (134 km^2^; 1,762 mm).

Model odds ratios, which represent the change in the response variable (in terms of odds) resulting from a one-unit change in a given predictor variable^17^, indicated that independent of humans and invasive mammals, smaller island size was associated with greater extirpation risk for non-volant mammals and volant birds, while amphibians, reptiles, bats and non-volant birds had greater extirpation risk on larger islands (Tables 1 and 2; Fig. 1a). Reptiles, non-volant mammals, and bats had greater extirpation risk on drier islands, and amphibians and non-volant birds had greater risk on wetter islands (Tables 1 and 2; Fig. 1a).

### Effects of humans and invasive mammals on extirpation risk

To understand the impacts of each invasive mammal group, we determined the characteristics of islands on which they occurred. Fifty-four percent of the islands in our data were uninhabited by humans; most uninhabited islands either lacked invasive mammals (46%) or contained only rats and/or cats (45%). Uninhabited islands were small and dry relative to the full set of islands (median area for uninhabited islands=0.6 km^2^ versus all islands=9.7 km^2^; median annual precipitation for uninhabited islands=907 mm versus all islands=1,326 mm). On uninhabited islands, the presence of rats increased modelled extirpation risk particularly for amphibians, reptiles, volant birds and non-volant mammals, while the presence of cats strongly increased risk for non-volant birds. Rats and cats had synergistic (but not strictly additive) effects on extirpation risk when they co-occurred on islands (Supplementary Fig. 2).

Islands inhabited by people were generally larger and wetter than uninhabited islands (median area=134 km^2^; median precipitation=1,762 mm). Six common combinations of invasive mammals occurred on 80% of the inhabited islands in the data: (1) rats only; (2) rats and cats; (3) rats and pigs; (4) rats, cats and pigs; (5) rats, cats and mustelids/mongooses; and (6) rats, cats, pigs and mustelids/mongooses (that is, all the invasive mammal types included in the final model). Model odds ratios showed that the presence of humans, rats and mustelids/mongooses increased extirpation risk consistently for all native vertebrate groups, while cats and pigs affected native groups differently (Table 2; Fig. 1b).

### Predictions of extirpation risk across island conditions

**Figure 2:**
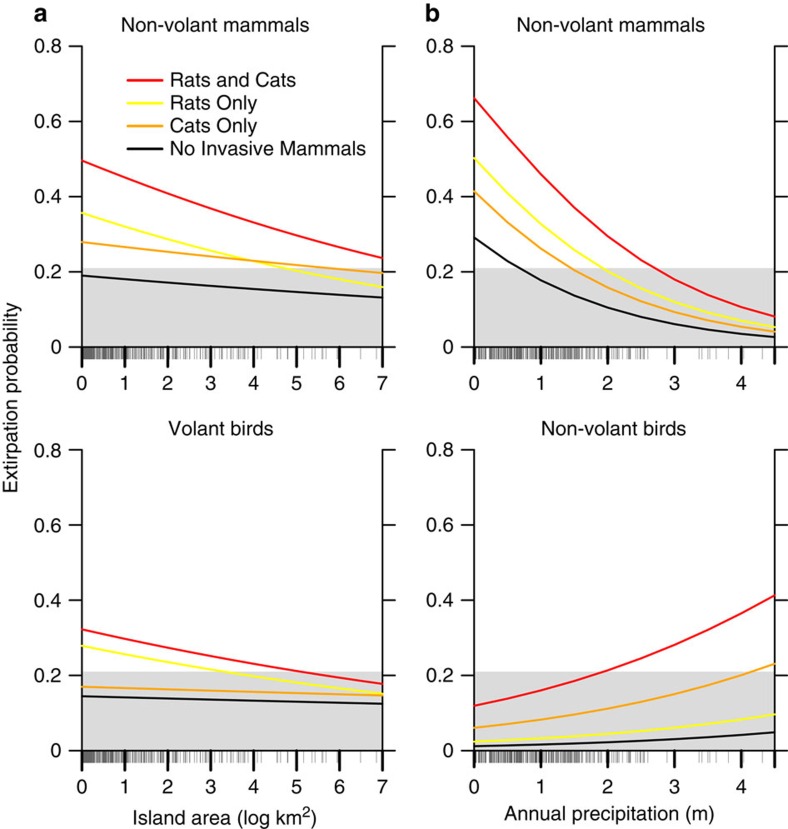
Modelled extirpation probabilities on uninhabited islands with invasive rats and cats. Background colour represents the predicted persistence (grey) or extirpation (white) of native island populations (Supplementary Table 5). Line colours correspond to the invasive mammal type(s) used to generate each set of model predictions: no invasive mammals (black), cats only (orange), rats only (yellow) or cats and rats (red). (**a**) Modelled extirpation probabilities for non-volant mammals and volant birds across the range of island areas for uninhabited islands in the data set, with precipitation held at the median value for uninhabited islands lacking invasive mammals or containing only rats and/or cats (907 mm). (**b**) Modelled extirpation probabilities for non-volant mammals and non-volant birds across the range of precipitation values for uninhabited islands in the data set, with island area held at the median for uninhabited islands lacking invasive mammals or containing only rats and/or cats (0.6 km^2^). Rug plots on *x* axes correspond to all area (**a**) or precipitation **(b**) values for island-species records on these islands. Standard errors for the predictions were calculated from K-fold cross-validation (not shown because are too small to appear in graphs; minimum s.e.=0.0002, maximum s.e.=0.0158).

**Figure 3:**
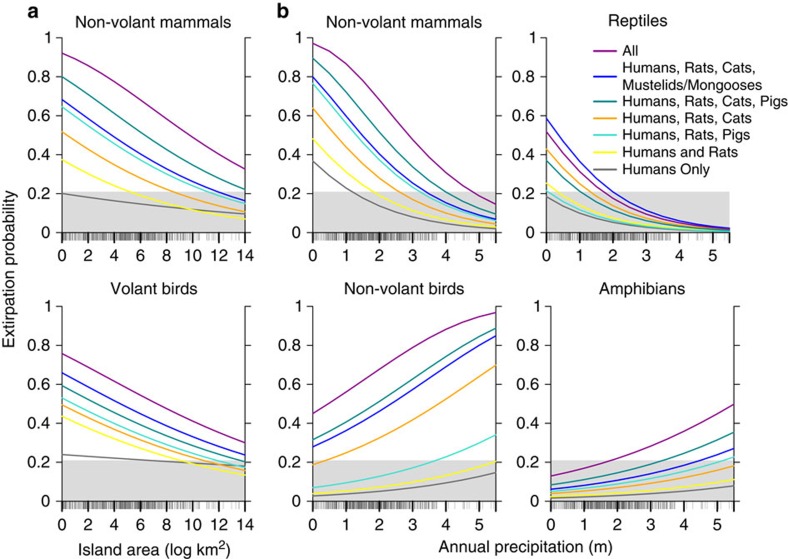
Modelled extirpation probabilities on inhabited islands with invasive mammals. Background colour represents the predicted persistence (grey) or extirpation (white) of native island populations (Supplementary Table 5). Line colours correspond to the invasive mammal type(s) used to generate each set of model predictions for inhabited islands: invasive mammals absent (black); humans and rats (yellow); humans, rats and pigs (light turquoise); humans, rats and cats (orange); humans, rats, cats and pigs (dark turquoise); humans, rats, cats and mustelids/mongooses (blue); humans and all invasive mammal types (magenta). (**a**) Modelled extirpation probabilities for non-volant mammals and volant birds across the complete range of island areas in the data set, with precipitation held at the median value for inhabited islands (1,762 mm). (**b**) Modelled extirpation probabilities across the complete range of island precipitation values for non-volant mammals, non-volant birds, reptiles and amphibians, with area held at the median value for inhabited islands (134 km^2^). Rug plots on *x* axes correspond to the area (**a**) or precipitation (**b**) values for all island-species records on inhabited islands. Standard errors were calculated from K-fold cross-validation (not shown because are too small to appear in graphs; minimum s.e.=0.0002, maximum s.e.=0.0202).

We examined the combined effects of different variables on extirpation risk by comparing model-fitted values across the range of each covariate (Figs 2 and 3 and Supplementary Figs 3 and 4). Rat impacts depended on island size, with stronger impacts to a subset of native groups on small islands (Tables 1 and 2; Figs 2a and 3a). For all native groups, co-occurrence with more invasive mammal types led to greater extirpation risk; however, the threat from different combinations was context-specific and was a function of native species traits, island characteristics and the suite of invasive mammals present (Figs 2 and 3 and Supplementary Figs 3 and 4).

We determined a logistic threshold for translating model-predicted continuous probabilities into predictions of population extirpation or persistence^17^, and tested additional thresholds to assess the sensitivity of the results to threshold choice (Supplementary Table 5). Based on a threshold associated with an 80% true-positive rate, the presence of invasive mammals on islands shifted the prediction for many native populations from likely persistence to likely extirpation (Figs 2 and 3 and Supplementary Figs 3 and 4).

### Predictions of extirpation risk for extant populations

We used the model fitted values and logistic threshold to predict extirpation or persistence for the 1,998 extant threatened vertebrate populations in our data. In the absence of conservation interventions, the model predicted extirpation for 896 populations (45%). The group most at risk was volant birds (extirpation predicted for 67% of populations), followed by non-volant mammals (44%), amphibians (43%), bats (34%), reptiles (21%) and non-volant birds (17%). At the maximum false-positive rate (the worst-case for model performance), we predicted at least 475 extirpations (24%). We were unable to predict the timing of these extirpations due to insufficient temporal data (Supplementary Fig. 5). Nearly half the predicted extirpations (*n*=401) are populations of species that are restricted to a single island; for these species, island extirpation would equate to global extinction.

### Predicted conservation benefits of managing invasive mammals

Seventy-nine percent of extant threatened vertebrate populations co-occur on islands with invasive rats, cats, pigs, and/or mustelids/mongooses. To quantify the conservation impact of managing these invasive species, we recalculated model predictions assuming they were eradicated from all islands where they co-occur with threatened species. Assuming no false-positive extirpations, eradication could prevent 75% of predicted extirpations (*n*=669). Under the maximum false-positive rate (475 predicted extirpations), eradication could prevent 41% of extirpations (n=194).

## Discussion

It is well known that invasive mammals cause endangerment and extinction of island species^5^. However, a nuanced and quantitative understanding of their global impacts has been unavailable due to a lack of comprehensive data. Our study quantifies patterns that were unknown previously or were only hypothesized, including (1) identification of the invasive mammals most strongly associated with island extirpations of threatened species globally, (2) detailed insights into these species' impacts on different native taxa in different island environments, and synergistic effects between abiotic conditions on islands and the effects of invasive mammals on native species; (3) interactions among multiple invasive mammal types on a single island, across island abiotic conditions, and (4) quantitative predictions of reductions in extinction risk expected for individual threatened species, resulting from the eradication of invasive mammals.

Four of the twelve invasive mammal groups we analysed—rats, cats, pigs and mustelids/mongooses—accounted for most of the variation in threatened vertebrate extirpations on islands. Rats and cats are commonly considered among the worst mammalian invaders on islands worldwide^4^,^5^, and our results provide further evidence for their widespread impacts on island species globally. In contrast, pigs, mustelids and mongooses are known to affect island species and ecosystems in some situations^18^,^19^, but their global impacts are less often considered. The other invasive mammal groups in our analysis—especially goats, mice, and rabbits—are known to impact island species and ecosystems in some contexts^20^,^21^,^22^, but our results suggest they are not the most important drivers of extirpation risk at a global scale. Analysis of extirpation patterns at the scale of individual archipelagos or regions would help determine the magnitude of these other groups' impacts on particular ecosystems and species, ideally including those we were unable to include in this analysis due to insufficient data (plants and invertebrates).

The effects of each invasive mammal on extirpation risk were a result of interactions between native vertebrate groups, island conditions and invasive mammal combinations. Our analysis identified a small number of interactions that explained most of the variation in extirpation patterns (Table 2). Rats were the only invasive mammal group whose impacts depended on island area, with stronger impacts on certain native taxa on small islands. Newly introduced rat populations on islands can undergo rapid population growth^23^; on small islands in particular, this could lead to dense rat populations and a faster depletion of native species' populations. Larger islands are more likely to contain refugia where native species can avoid predation or competition from invasive rats^8^,^9^. Larger islands are also more likely to contain native rodents or other native terrestrial predators, which may compete with or predate upon invasive rats, thus limiting their population sizes and reducing their impacts on native species. Finally, the presence of native rodents or other predators on larger islands may be associated with native species that have evolved better defenses against predation.

When rats and cats occurred together on islands, they had synergistic effects on extirpation risk, and the strength of the synergy was a function of both native group and island conditions. Specifically, model-predicted extirpation risk for most native species groups was higher with both rats and cats present on islands than with rats alone; the degree to which this was the case varied across native groups, island area and precipitation. This result is not consistent with mesopredator suppression theory, which predicts lower extirpation rates on islands containing both apex and mesopredators (that is, cats and rats) compared with islands with only a mesopredator (for example, rats only)^24^. The presence or absence of mesopredator suppression on a given island is likely a result of many complex and interacting ecological factors, including seasonality of resources, the presence and abundance of alternative prey (either native or introduced), and behavioural responses of apex and mesopredators^24^,^25^,^26^,^27^. The strength of mesopredator suppression effects has been shown to vary spatially even within a single island^28^. Strong evidence for mesopredator suppression and reduced extirpation risk for native species has been found in island systems in Australia and New Zealand^6^,^28^; however, studies on islands in the Mediterranean and the Western Indian Ocean found no evidence for this phenomenon^25^,^29^, and a global review found that cat impacts on native birds were greater when introduced prey species were present^4^. At a global scale, our results suggest that mesopredator suppression is certainly not ubiquitous and may be relatively uncommon.

Invasive pigs have direct and indirect impacts on native species, and are known to alter plant communities and ecosystem processes through their omnivorous foraging behaviour^30^. Our results suggest that native bats are particularly vulnerable to impacts from invasive pigs across all island conditions. Threatened bat species in our data may be especially dependent on intact habitat for survival and reproduction, and may thus be more vulnerable than other native taxa to habitat modification^31^. Most of the other native taxa are also vulnerable to impacts from invasive pigs; we speculate that the majority of these impacts result from habitat modification, though pigs also prey on ground-dwelling vertebrates and compete with native species for resources^30^.

The strength of mustelid/mongoose impacts here is remarkable given their presence on only 11% of the islands in our data. This result may be explained partly by mustelid/mongoose introductions being relatively recent compared with those of other groups^32^,^33^, and more recent extirpations being better documented. Like rats, mustelids/mongooses in our analysis had consistent impacts on all native vertebrate groups. Both rats and mustelids/mongooses are small- to medium-sized generalist omnivores that can relatively easily access small vertebrate prey. In contrast, the varied impact of cats on different native groups may be due to their purely carnivorous diets, their ability to survive on resource-poor islands^34^, or their wider distribution on islands globally compared with mustelids/mongooses.

As we predicted, the presence of human populations on islands increased extirpation risk for all native groups. Permanent human settlements are associated with myriad anthropogenic threats to native species. In addition to direct exploitation by humans, loss of native habitat is inevitable on inhabited islands, as people use land for dwellings, agriculture, transportation and other infrastructure. Pollution is also more likely to occur on inhabited islands, as humans disperse biological as well as toxic waste products and pesticides throughout the environment. Permanent human populations exacerbate threats from invasive mammals due to increased numbers of intentional and accidental introductions, escape of domesticated animals which become feral, and modification of habitats in ways that benefit invasive species. Our analysis is unable to distinguish between these different anthropogenic effects, but it is reasonable to conclude that native species on most of the inhabited islands in our data are subject to one or more of these threats in addition to the impacts of invasive mammals.

The influence of island geography and climate on both intrinsic extirpation risk and invasive mammal impacts is not well understood beyond the predictions and extensions of island biogeography theory (IBT)^8^,^35^ and some case studies^10^. Our finding of greater extirpation rates on small islands for non-volant mammals and volant birds is consistent with IBT^35^ and with the large number of bird and mammal extinctions historically documented on small oceanic islands^9^,^36^. Unexpectedly, other native groups had greater extirpation risk on large islands. IBT suggests that native populations on large islands should be less inherently vulnerable to stochastic events such as storms and disease outbreaks that could cause extirpation. There are several possible explanations for this surprising result. First, the assumptions of IBT may not fully apply to the native populations in our study because we focused on a nonrandom subset of species (extinct and threatened vertebrates) rather than the full suite of species on each island. Second, many native species extirpations had already taken place before the timeframe of our data set; our data thus represent populations, species, and ecosystems that function differently than they did before human colonization and associated prehistoric extinctions^37^,^38^. Third, human-inhabited islands in our data were generally larger than uninhabited islands. The taxa that exhibited higher extirpation risk on larger islands—amphibians, reptiles, bats and non-volant birds—may be more susceptible to anthropogenic impacts including habitat destruction, pollution and poaching. Under this explanation, island area represents a suite of anthropogenic threats not fully captured by the human presence/absence variable included in our model. Finally, because larger islands tend to have more people, they may simply experience more intensive biological surveys such that native populations and their subsequent extirpations were more likely to be recorded. This explanation is especially plausible for less thoroughly studied or more elusive taxa such as amphibians, reptiles and bats, which may be more difficult to record on occasional survey visits to small islands.

We found no effect of island size on extirpation risk for amphibians, reptiles, non-volant birds, or bats in the presence of any invasive mammal combination. Amphibians, reptiles and non-volant birds are less mobile than the other native groups, which may make them more vulnerable both to predation by invasive predators and to habitat disturbance by humans and invasive pigs. Bats are highly mobile, but our results indicate they are severely threatened by invasive pigs and human populations across all island sizes. As discussed above, bats may be particularly susceptible to changes in their habitat, even on very large islands. These results are consistent with research suggesting that habitat loss is the leading threat to island bat species globally^31^.

To our knowledge, the effects of precipitation on island population extirpations or invasive mammal impacts have never been explicitly investigated at a global scale. Our finding of greater extirpation risk for mammals (non-volant mammals and bats) on drier islands is consistent with the results of a regional-scale analysis of mammal extinctions on Australian islands^6^. Several hypotheses may explain this as well as the varied effects of precipitation on different native groups in our analysis: (1) greater productivity on wet islands may lead to less variation in population size or greater availability of refuges from predation, (2) some invasive mammals, particularly cats, are particularly well adapted to dry conditions^34^, potentially leading to higher population densities and stronger impacts on some islands, and (3) impacts from invasive species not included in our final model (for example, other mammals, invertebrates and plants), or from other anthropogenic threats, may be associated with higher or lower precipitation. The seasonality of precipitation may also play a role in extirpation patterns; for example, highly seasonal rainfall could lead to wider annual population swings for native species, which in turn could lead to increased extirpation risk during dry, resource-limited periods. This effect may be exacerbated by increased competition or predation pressure from invasive mammals^10^. Our analysis was unable to test between these non-mutually exclusive and potentially interacting hypotheses, and the role of precipitation in extirpations remains an important avenue for further study.

Non-volant mammals and reptiles in our analysis were more vulnerable to all types of invasive mammals on drier islands. This result is consistent with our hypothesis that drier islands are generally resource-poor, potentially leading to stronger predation pressure on native species and more competition for limited resources. However, we found the opposite pattern for non-volant birds and amphibians, which were more vulnerable to invasive mammals on wetter islands. A potential explanation for this result is that non-volant birds and amphibians may be more restricted to wetter habitats and associated resources, which would make them more vulnerable to the impacts of predation, competition, and habitat modification from invasive mammals in such environments.

In addition to providing a more nuanced understanding of the drivers of island extirpations than has been available previously, our model results can be used to predict the potential outcomes of conservation interventions. We provide support for some common assumptions: for example, three of the four invasive mammal groups most often targeted by eradication efforts—rats, cats, and pigs^39^—are, indeed, globally important drivers of extirpation risk and managing (that is, controlling or eradicating) them will help conserve threatened vertebrate species^40^. Similarly, managing mustelids and mongooses is a primary conservation concern in regions where these species have been introduced^19^,^32^, and our results strengthen the evidence for continuing such efforts.

Our results also reveal some surprising predictions about management outcomes. Just as we can quantify the extirpation risk for insular species based on the interacting factors in our model, we can also quantify the potential benefit of managing invasive mammals. If the ecology of an island system is such that invasive mammals have little impact on native species—due to the mechanisms hypothesized above as well as any number of other ecological mechanisms acting at the scale of single islands—managing the invasive mammals may not provide the intended conservation benefits. For example, because our model indicated that, in general, non-volant mammals and reptiles are only weakly affected by invasive mammals on wetter islands, managing the invasive mammals on such islands may lead to only minor benefits for these groups. Similarly, non-volant birds and amphibians in our analysis are less susceptible to invasive mammal impacts on drier islands, suggesting that managing invasive species to conserve these native groups may be more effective on wetter islands. Finally, while non-volant mammals and volant birds are predicted to benefit from invasive mammal management on islands of all sizes, the benefits may be substantially greater on small islands. In all these cases, it is important to note that invasive mammal impacts and management outcomes are site- and ecosystem-specific and may be difficult to predict^41^,^42^. Careful assessment of the potential outcomes is needed in specific cases before any management actions are implemented. Our analysis identifies global-scale patterns that should provide a foundation for more detailed investigations and predictions at the scale of single islands and archipelagos.

Our analysis and its implications for management should be considered in light of a long history of human and invasive mammal impacts on islands. Our data set includes native vertebrate populations that were recorded on islands up to 500 years before the present. At that time, humans and invasive mammals had already occupied many islands around the world for hundreds, thousands, and in some cases tens of thousands of years, and had greatly altered island ecosystems^37^,^38^. Many of the insular species that were most vulnerable to poaching, habitat loss, and invasive mammal impacts were already extinct or had been reduced to small remnant populations by 500 years before the present. For example, at least 2,000 bird species were lost in the tropical Pacific in prehistoric times^37^, and about 27% of insular endemic mammals are thought to have become extinct after the arrival of humans^36^. Our data therefore represent subsets of the original suite of species present on each island, and ecosystems that function differently than they did before human colonization^38^. Islands that were colonized more recently may contain a more complete set of original species, and differences in colonization history likely increase the variability in our estimates of human and invasive mammal impacts. In addition, our results may underestimate the role of humans and invasive mammals in driving extirpations, since we did not include prehistoric extirpations that were almost certainly caused by these factors before the timeframe of our data. Nevertheless, since these initial waves of extinctions had already taken place on most islands by the time our data set begins, our model is likely a more accurate predictor of the impacts of current and future management actions than it would be if we were able to incorporate prehistorically extinct species into the data set.

While many island species and populations were extirpated in prehistoric and recent times, many more persist to the present day despite ongoing threats. Some extant species may be relatively resistant to anthropogenic impacts, but others are small remnant populations that may persist only because of a more recent invasion history on their breeding islands or the availability of habitat refuges. These latter populations face a high likelihood of extinction and represent extinction debt that we may pay in the future if we fail to implement appropriate conservation actions^43^. Our model enables the identification and quantification of this extinction debt. Specifically, we can compare model predictions for an extant population under current conditions (that is, invasive mammals present) to the predictions under potential management scenarios (that is, invasive mammals controlled or removed) to quantify the extinction risk, or debt, that would be eliminated via management actions. Populations that stand to benefit the most from managing invasive mammals are those for which we can most substantially reduce extinction debt. The global potential for reducing extinction debt by managing invasive mammals is presented in Supplementary Table 5.

Our analysis adds quantitative support to the assertion that managing invasive mammals on islands is an essential tool for conserving global biodiversity^40^. More than 800 successful invasive mammal eradications have been implemented to date^40^, and eradications are being conducted on increasingly large and complex islands^44^. Other valuable interventions include the creation of invasive mammal-free reserves in fenced enclosures, peninsulas or small offshore islands to which threatened species could be translocated^45^,^46^, and importantly, prevention of invasive mammal incursion (or reincursion) to avert potential additional extirpations^47^. Many of these actions have succeeded in restoring and conserving threatened species^40^. Nevertheless, decisions about the invasive mammals and islands on which management actions should be focused have been based on observed or assumed threats from particular mammals^20^,^48^, and/or on island-specific conservation needs^49^,^50^. We present the largest empirical test to date of many biological assumptions that often guide such management decisions. We identify the most damaging invasive mammals to native island vertebrates at a global scale, and highlight situations in which managing them should provide substantial conservation gains. Importantly, we also identify contexts where such interventions may not provide the intended benefits and may be a less effective conservation strategy. Such data-driven, quantitative approaches are underutilized in conservation, yet are urgently needed to allocate limited conservation resources strategically to maximize benefits for threatened biodiversity.

## Methods

### Native and non-native island species data set

The core data set in our analysis was the Threatened Island Biodiversity Database Version 2012.1 (ref. 13), which contains current and historic island distributions for all terrestrial vertebrates classified as Endangered, Critically Endangered, Extinct in the Wild, or Extinct on the IUCN Red List^14^. The data set identifies whether each species currently persists on or has been extirpated from each of its historic breeding islands, with extirpation defined as the disappearance of a species from a single island after AD 1500 (see Supplementary Methods and ref. 51 for details). We determined the flight ability (volant versus non-volant) of all threatened species, and the mean body mass of species for which data exist (Supplementary Methods and Supplementary Data 2). We classified native species into six groups based on class and flight ability (henceforth class/volancy): volant birds, non-volant birds, bats (that is, volant mammals), non-volant mammals, amphibians and reptiles. Along with body mass, flight ability has been shown to be a primary determinant of extinction risk for insular birds and mammals, with non-volant species being particularly vulnerable to predation by both invasive mammals and humans^33^,^36^,^37^ (Supplementary Methods). Because flight ability as a covariate only applies to two of the four terrestrial vertebrate classes, including it in the analysis required combining class and volancy into a single categorical variable.

The Threatened Island Biodiversity Database also includes information about invasive species known or suspected to be present on each island; we used this information to record the presence or absence of all non-native mammals on islands for which we could find information^13^,^51^ (see Supplementary Methods and ref. 51 for details). If a non-native mammal was considered likely but not confirmed on an island, we classified it as present. If there was no information about a given invasive mammal on an island, we classified it as absent. This assumption likely led us to misclassify some species as absent when they were actually present—particularly rodents, which can be difficult to detect. Thus, our analysis may underestimate the impacts of some invasive mammals. We classified as ‘present' all non-native mammals that have been eradicated from islands^52^, based on the assumption that these species existed on these islands long enough to impact native vertebrate populations; we removed this classification to predict the current and future impacts of invasive mammals on extant native populations. We included eradications that took place as long ago as 1630, but most eradications in our data set took place after 1970.

We identified ∼160 invasive mammals that had become established on islands and classified them into 12 broad groups (Supplementary Methods and Supplementary Table 2). We sought a balance between maximizing the representation of each group across islands (that is, sample size within strata) and considering as many groups as needed to distinguish the impacts of different invasive mammals.

### Island attributes data set

We obtained island areas from the Global Islands Database^53^ and elevation and climate data from the BioClim data set (resolution 30 s; ref. 54; Supplementary Methods). We created using Geographic Information Systems (GIS) overlays of our island data set and each abiotic covariate (Table 1). We developed a single estimate for each climatic variable per island by calculating the arithmetic mean of the center points of all raster grid cells located within the boundary of an island polygon. We supplemented missing area, climate and elevation GIS data with external data sources, values from nearby islands, or linear interpolation based on values for nearby islands (Supplementary Methods). We determined whether people likely maintain permanent settlements on each island (Supplementary Methods).

### Extinction probability analysis

We constructed logistic models to examine global patterns of island population extirpation and persistence for threatened vertebrate species. We included each covariate listed in Table 1, as well as interactions for which we had specific hypotheses regarding their effects on extirpation patterns (Table 2). The binary response variable in our analysis was the extirpation or persistence of a native species on one island. Specifically, each native threatened species on an island constituted a data point, in which the species is either extinct on the island (1=an extirpation event) or it persists there (0=no extirpation event). We were unable to include time as a factor in our analysis because only about half the extirpation records in our data had timing information, and the range of values for these records was too wide to be informative (Supplementary Methods and Supplementary Fig. 5).

Our analysis was based on island-level co-occurrences between threatened species and invasive mammals. For example, a threatened reptile species on an island with invasive rats and mice is assumed to co-occur with, and potentially be impacted by, both invasive species. If the reptile is extinct on the island, rats and mice are implicated in its extirpation. The geographic and temporal scale of our study was too large to allow for more detailed analyses of the direct and indirect impacts of invasive mammals at finer spatial scales, including but not limited to: predation, competition, habitat modification, disease introduction or transmission and changes in the abundance of other interacting species.

We built logistic models using generalized estimating equations (GEEs), which are similar to generalized linear models but include an additional variance component that accommodates clustered data^15^. GEEs are appropriate for this analysis because our data were clustered by island: extirpation rates may exhibit autocorrelation at the island level (for example, due to stochastic storm events or disease outbreaks) that is not accounted for by variables we measured. We fit models to the data using the R package *geepack*^55^. We specified a binomial distribution with a complementary log-log link function^17^,^56^ because the response data set was asymmetric, made up of mostly zeros (that is, population persistence). The grouping structure was the island and we used an exchangeable working correlation matrix^57^. We used log transformations of continuous variables that had skewed distributions (Table 1).

We calculated pairwise Pearson correlation coefficients between all continuous explanatory variables, as well as the ordinal variable human population index to test for correlations between these covariates. Due to a strong correlation (Pearson's *r*>0.7; ref. 58) between area and elevation, we used the residuals from the area-elevation relationship as a covariate in the analysis instead of island elevation. The area-elevation relationship of an island is associated with ecosystem diversity, which in turn can influence native population dynamics and the outcomes of species invasions^59^. To account for differences in island elevation independent of area, we obtained the residuals from the area-elevation relationship, which represent the independent portion of the variation in elevation. We used these residuals as a covariate in the GEE instead of island elevation.

We examined associations (that is, correlations) between occurrences of different introduced mammal groups on islands using non-metric multidimensional scaling. In this analysis, clusters represent the grouping of explanatory variables (introduced mammal groups) in relation to themselves and not the grouping of these variables in their relation to the response variable (native species extirpation rates)^60^. We constructed a multidimensional scaling ordination based on a Euclidian distance matrix generated from the presence and absence of invasive taxa (columns) among all the islands (samples). We identified no strong relationships among introduced taxa and, therefore, kept all the mammal groups in the analysis.

We began the model selection procedure with 20 main effects (Table 1; as described above, island elevation was replaced by the area-elevation residuals). To reduce the initial set of covariates, we excluded the four invasive mammal groups that occurred on <10% of the islands: primates, medium-sized omnivores, small omnivores and medium-sized herbivores. We also excluded the body mass of threatened species due to a large number of missing values. However, we ran the model selection procedure on the subset of data for which we did have body mass data, and this variable was not selected in any of the top-weighted models, indicating that excluding this variable did not affect the results (Supplementary Methods).

We built a preliminary main-effects model with the remaining 15 variables. For each model order (that is, from 1 to 15 covariates), we ran all possible models and identified the highest weighted model(s) based on QICu using the R package *MuMIn*^61^. QICu is similar to Akaike's information criterion but is specific to GEEs and is used to rank models that have the same pre-specified correlation structure^15^. The number of equally weighted top models (models with ΔQICu<2) for each order ranged from one to four. We used the highest weighted model, or model-averaged coefficients from the 2–4 top models, to generate fitted values and calculate mean square error (MSE)^16^ for each model order. We used MSE to evaluate model fit because GEEs are not likelihood-based and do not have associated deviance measures. There was a trend of diminishing returns such that model sensitivity improved with increasing model order up to the seventh order, but only minimally above this (Supplementary Fig. 1). We chose the highest weighted seventh-order model as the preliminary main effects model, which contained the following terms: class/volancy, island area, annual precipitation, human presence/absence, and introduced cats, pigs and mustelids/mongooses.

Next, we tested interactions between these seven covariates that we hypothesized a priori to be potentially important in driving island extirpation patterns (Table 2). We retained interaction terms that produced a decrease in QICu of >10—a conservative limit for determining model improvement^62^. In this way, four terms—interactions between class/volancy and area, precipitation, cats, and pigs—were added to the model. Rats did not appear in the preliminary main effects model, but we tested their addition to the model because (1) they are widely recognized as one of the most destructive mammalian invaders on islands globally^5^, and (2) they are the focus of most island mammal eradication efforts^39^. We found that the addition of rats and the rat*area interaction term decreased QICu by >20, so we added these terms to the model. The final model thus included eight main effects and five interaction terms (Supplementary Table 3).

### Model validation and assessment

We generated observation-level and cluster-level measures of influence using SAS statistical software^63^, and used deletion diagnostics to assess the contribution of each outlying observation or cluster to overall model fit and coefficient estimates. We used K-fold cross-validation to calculate four model evaluation statistics for the final model: mean prediction error, mean absolute prediction error, standard deviation of the prediction error and mean square error^16^ (Supplementary Table 4). For GEE K-fold cross validation, the clusters (that is, islands) in the data set were randomly divided into K groups (*K*=10). Each of the 10 groups was iteratively removed from the data set; the nine remaining groups were used to calibrate a new model, and the Kth group was used to generate evaluation statistics, construct a receiver operating characteristic curve, and calculate the AUC (see below). We repeated the K-fold validation process 1,000 times, resulting in 10,000 validation runs that we used to calculate model errors and AUC.

We assessed the predictive performance of the final model by calculating the AUC for each K-fold validation run. The AUC is a commonly used statistic for evaluating a logistic model's ability to correctly predict binary outcomes in the response variable based on the continuous probabilities in the model's fitted values^17^. An AUC of 0.5 indicates that a model is no better than random at predicting outcomes, while an AUC of 1 means it predicts outcomes perfectly. We assessed the robustness of model prediction performance by calculating the mean, standard deviation, and standard error of model AUC values across the 10,000 validation runs.

Receiver operating characteristic curves represent the tradeoff between a model's sensitivity (the true-positive rate (TPR); the ability to correctly identify extirpations) and its specificity (the true-negative rate (TNR); the ability to correctly identify persistence). Unless the AUC for a model is 1, an increase in the TPR entails a cost of a higher false-positive rate (Type II errors). Threshold values for binary prediction are chosen based on the cost of making Type I versus Type II errors. Given the conservation context of our study and the need to accurately determine extinction risk for island populations, we viewed false-negatives (missing a true extinction) as being more costly than false-positives (misidentifying a rare species as extinct). Thus, for the primary analysis we chose a threshold with an associated TPR of 80% (TNR=53%); we also examined two additional threshold values to assess the sensitivity of the results to threshold choice (Supplementary Table 5).

### Data availability

The data that support the findings of this study are included in Supplementary Data files 1 and 2. A subset of sensitive species distribution records have been removed from these data files in accordance with our agreement with specialists who provided the data. The raw (that is, unprocessed) data used in this study are contained in the Threatened Island Biodiversity Database (tib.islandconservation.org) and the BioClim data set^54^. Source data for Supplementary Fig. 5 are available from the corresponding author (E.E.M.).

## Additional information

**How to cite this article:** McCreless, E.E. *et al*. Past and estimated future impact of invasive alien mammals on insular threatened vertebrate populations. *Nat. Commun.* 7:12488 doi: 10.1038/ncomms12488 (2016).

## Supplementary Material

Supplementary Information Supplementary Figures 1-5, Supplementary Tables 1-5, Supplementary Methods and Supplementary References

Supplementary Data 1 Complete dataset, including all variables included in model selection. Sensitive species distribution have been removed at the request of experts who provided data.

Supplementary Data 2 Body mass data and associated references for threatened and extinct insular vertebrate species. Dataset includes each species' minimum and maximum body mass, when available, or body length (amphibians and reptiles) when mass data were unavailable. Also includes the average or estimated mass value that was used in the analysis for each species.

## Figures and Tables

**Table 1:** Abiotic and biotic covariates, hypotheses, inclusion in or exclusion from model and model results.

	**Covariate**	Hypothesized relationship (+/–) and mechanism driving native species extinction risk*	**Included in model selection**	**Included in final model**	**Model result**
Island attributes	Island area (log)	Higher extirpation rates expected on smaller islands (−)^35^	Yes	Yes	Effect varies by class/volancy and invasive rat presence
	Max elevation (log)	Habitat heterogeneity creates refuges from anthropogenic impacts (−)^8^	Yes†	No	NA
	Annual mean temperature	Temperature affects resource availability (+/−)	Yes	No	NA
	Temperature seasonality (log)	Seasonality of climate affects resource availability (+/−)	Yes	No	NA
	Total annual precipitation	Precipitation affects resource availability (+/−)^6^,^10^	Yes	Yes	Direction and magnitude of effect varies by class/volancy
	Human presence	Anthropogenic impacts (for example, poaching, habitat destruction, pollution) drive extirpations (+)^37^	Yes	Yes	Increases extirpation probability
Native species	Taxonomy and ability to fly (class/volancy)^‡^	Taxonomy and flight ability influence native species' intrinsic susceptibility to anthropogenic impacts and effects of invasive mammals (+/−)^33^,^36^,^37^	Yes	Yes	Extirpation probability for each native group depends on island area, precipitation and invasive cats and pigs
	Body mass (log)	Large versus small species differ in vulnerability to impacts from invasive mammals (+/−)^6^	No	NA	NA
Introduced mammals present	Canids	Predation, competition (+)^65^	Yes	No	No
	Felids	Predation, competition (+)^4^	Yes	Yes	Increase extirpation probability; strength of effect varies by class/volancy
	Mustelids and mongooses	Predation, competition (+)^18^,^19^	Yes	Yes	Increase extirpation probability
	Pigs	Predation, competition, habitat modification (+)^30^	Yes	Yes	Increase extirpation probability; strength of effect varies by class/volancy
	Primates	Predation, competition (+)^66^	No	NA	NA
	Medium-sized omnivores	Predation, competition (+)^67^	No	NA	NA
	Small omnivores	Predation, competition (+)^65^	No	NA	NA
	Rats	Predation, competition (+)^5^	Yes	Yes	Increase extirpation probability; stronger effect on smaller islands
	Mice	Predation, competition (+)^21^	Yes	No	NA
	Large herbivores	Competition, habitat modification (+)^68^	Yes	No	NA
	Medium-sized herbivores	Competition, habitat modification (+)^69^	No	NA	NA
	Lagomorphs	Competition, habitat modification (+)^22^	Yes	No	NA

^*^For each covariate, column 2 describes the hypothesized mechanism and direction of impact (+/−) on extirpation probability, with references provided where applicable.

^†^Due to collinearity between island area and elevation, topographic complexity was included in the model as the residuals of the area-elevation relationship (see Methods).

^‡^Six native groups: volant birds, non-volant birds, bats (that is, volant mammals), non-volant mammals, amphibians and reptiles (see Methods and Supplementary Methods).

**Table 2:** Interactions between covariates, hypotheses, inclusion in or exclusion from model and model results.

**Interaction**	Hypothesis*	**Interactions included in final model**	**Model result**
Area*Invasive mammal type	Invasive mammals expected to cause more extinctions on small islands due to smaller native populations, fewer alternative resources for invasive mammals and fewer refuges from invasive mammal impacts^8^Invasive mammal groups differ in ability to successfully colonize small islands, depending on dietary flexibility and total resource needs^7^	Rat*area	Invasive rats increase extirpation probability for all native groups, with a stronger effect on smaller islands
Temperature or precipitation*Invasive mammal type	Invasive mammal impacts may be stronger on resource-poor islands, which may correlate with low precipitation and extreme high or low temperatures^70^Invasive mammal groups differ in the ability to successfully invade environments with different climates^18^,^34^	None	NA
Area*Native class/volancy	Native vertebrate groups may differ in their ability to disperse to and diversify on islands of different sizes, due to differences in dispersal ability, body size and home range size^36^,^37^These differences may influence the vulnerability of different native groups to anthropogenic impacts on islands of different sizes	Area*Native class/volancy	Non-volant mammals and volant birds have higher extirpation risk on smaller islands; amphibians, bats, reptiles and non-volant birds have higher extirpation risk on larger islands†
Temperature or precipitation*Native class/volancy	Native vertebrate groups differ in susceptibility to anthropogenic impacts in different climates depending on life history traits (for example, endotherm versus ectotherm, home range size, flight ability)^10^	Precipitation*Native class/volancy	Reptiles, non-volant mammals and bats have higher extirpation risk on drier islands; non-volant birds and amphibians have higher extirpation risk on wetter islands; precipitation has no effect on extirpation risk for volant birds†
Native class/volancy*Invasive mammal type	Native vertebrate groups differ in vulnerability to different invasive mammal groups depending on ecological overlap and predator/prey or competition relationships between native species and invasive mammals^7^	Native class/volancy*PigNative class/volancy*cat	Invasive pigs increase extirpation probability for amphibians, bats, non-volant mammals, non-volant birds and volant birds; Invasive cats increase extirpation probability for non-volant birds, amphibians, reptiles and non-volant mammals†
Native species body mass*Native class/volancy	Different-sized species within each native group differ in life history traits that influence their vulnerability to anthropogenic impacts^33^	None	NA
Native species body mass*Invasive mammal type	Native vertebrates of different sizes vary in susceptibility to invasive mammal impacts including predation (predators and omnivores) and habitat modification (omnivores and herbivores)^33^	None	NA

Abbreviation: NA, not applicable.

^*^For each interaction, column 2 describes hypothesized mechanisms by which the interaction would impact extirpation probability for threatened island species.

^†^Native groups are listed in order of the strength of the effect of the interaction, from largest to smallest.
