# Supplementary material for: Past and estimated future impact of invasive alien mammals on insular threatened vertebrate populations
Source: Nat Commun. 2016 Aug 18;7:12488. doi: 10.1038/ncomms12488 (PMC4992154; doi:10.1038/ncomms12488)
Supplement: Supplementary Information — Supplementary Figures 1-5, Supplementary Tables 1-5, Supplementary Methods and Supplementary References [file ncomms12488-s1.pdf]

Supplementary Figures

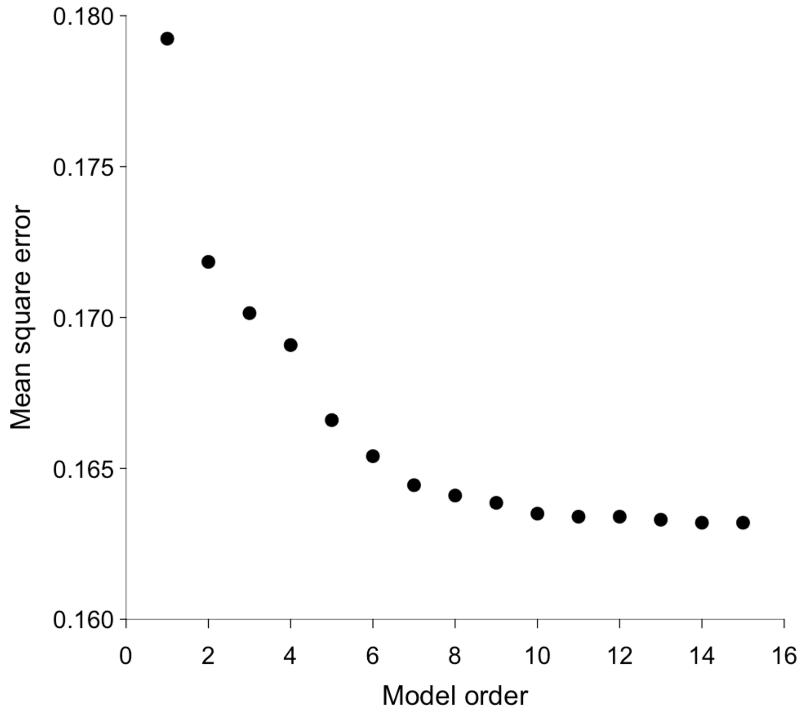

Supplementary Figure 1. Model order vs. mean square error. Model mean square error (MSE) calculated from the highest weighted model, or model-averaged coefficients from all equally weighted top models (ΔQICu<2), for each model order (one to 15 covariates) included in the model selection procedure.

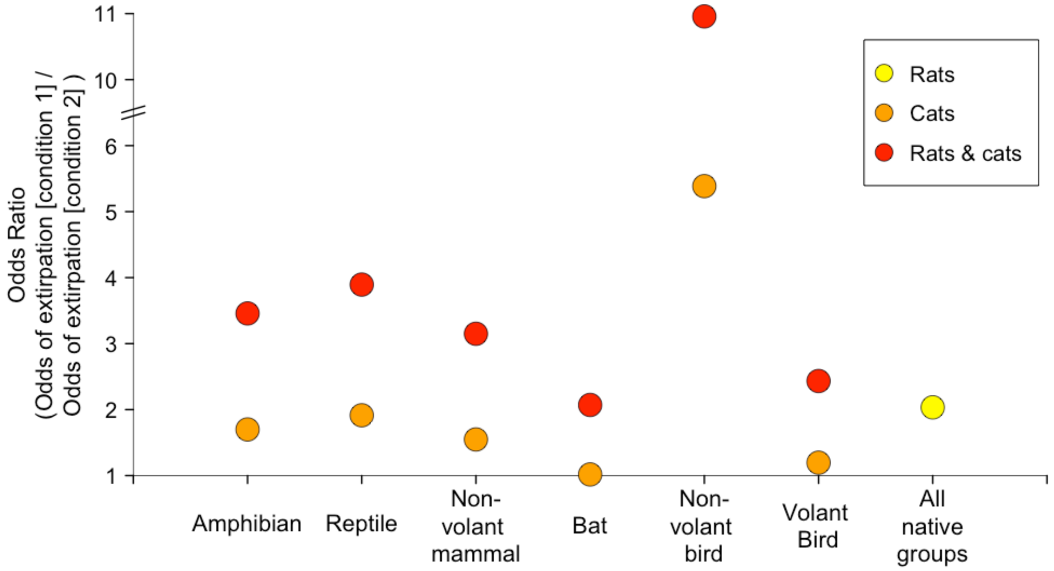

Supplementary Figure 2. Model-predicted odds ratios comparing extirpation risk due to rats and cats on uninhabited islands. Yellow: the ratio of the odds of extirpation on rat-infested vs. invasive mammal-free islands, for all native species groups. Orange and red: the ratios of the odds of extirpation on islands with cats only (orange) or rats and cats (red) vs. the odds on invasive mammal-free islands. Odds ratios were calculated with area and precipitation held at the median values for uninhabited islands (0.6 km^2; 907 mm).

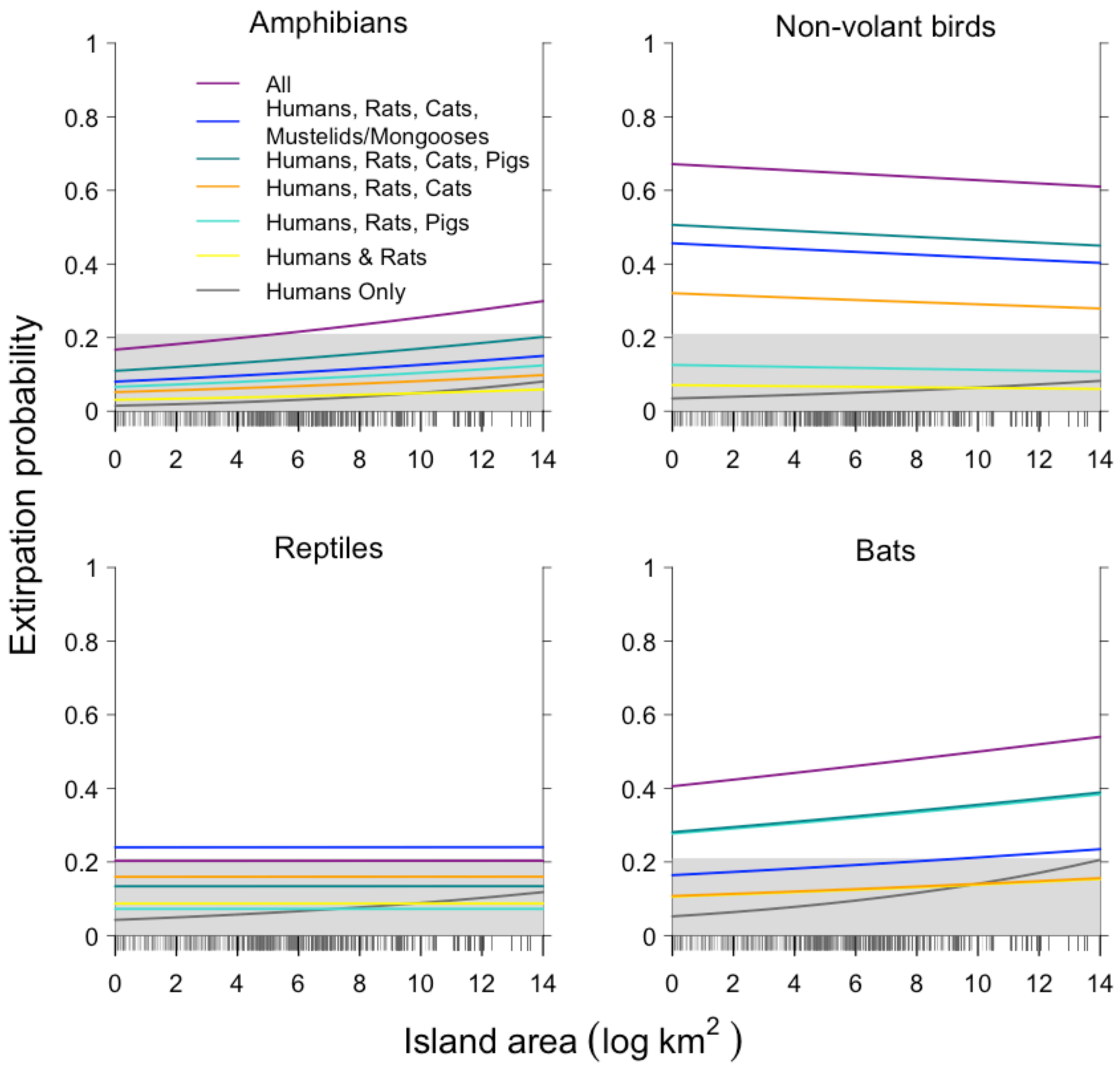

Supplementary Figure 3. Modeled extirpation probabilities across island areas on inhabited islands with invasive mammals. Background color represents the predicted persistence (gray) or extirpation (white) of native island populations (Supplementary Table 5). Colored lines represent model-predicted extirpation probabilities for amphibians, non-volant birds, reptiles, and bats across the complete range of island areas in the dataset. Line colors correspond to the invasive mammal type(s) used to generate each set of model predictions for inhabited islands: invasive mammals absent (black); humans and rats (yellow); humans, rats, and pigs (light turquoise); humans, rats, and cats (orange); humans, rats, cats, and pigs (dark turquoise); humans, rats, cats, and mustelids/mongooses (blue); humans and all invasive mammal types (magenta). Model fitted values were calculated with precipitation held at the median value for inhabited islands (1,762 mm). Rug plots on the x-axes correspond to island area values for all island-species records on inhabited islands. Standard errors for the predictions were calculated from K-fold cross-validation (not shown because are too small to be seen in the graphs; min SE=0.0005, max SE=0.0154).

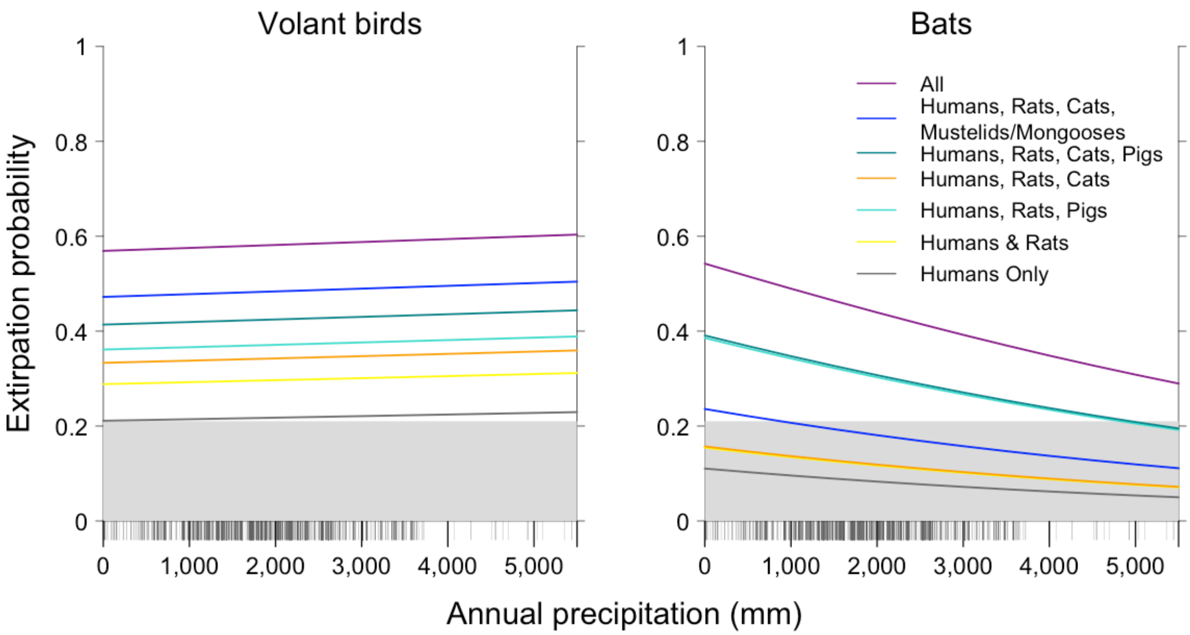

Supplementary Figure 4. Modeled extirpation probabilities across precipitation levels on inhabited islands with invasive mammals. Background color represents the predicted persistence (gray) or extirpation (white) of native island populations (Supplementary Table 5). Colored lines are the model-predicted extirpation probabilities for volant birds and bats across the complete range of precipitation values in the dataset. Line colors correspond to the invasive mammal type(s) used to generate each set of model predictions for inhabited islands: invasive mammals absent (black); humans and rats (yellow); humans, rats, and pigs (light turquoise); humans, rats, and cats (orange); humans, rats, cats, and pigs (dark turquoise); humans, rats, cats, and mustelids/mongooses (blue); humans and all invasive mammal types (magenta). Model fitted values were calculated with area held at the median value for inhabited islands (134 km^2). Rug plots on the x-axes correspond to precipitation values for all island-species records on inhabited islands. Standard errors for the predictions were calculated from K-fold cross-validation (not shown because are too small to be seen in the graphs; min SE=0.0015, max SE=0.0103).

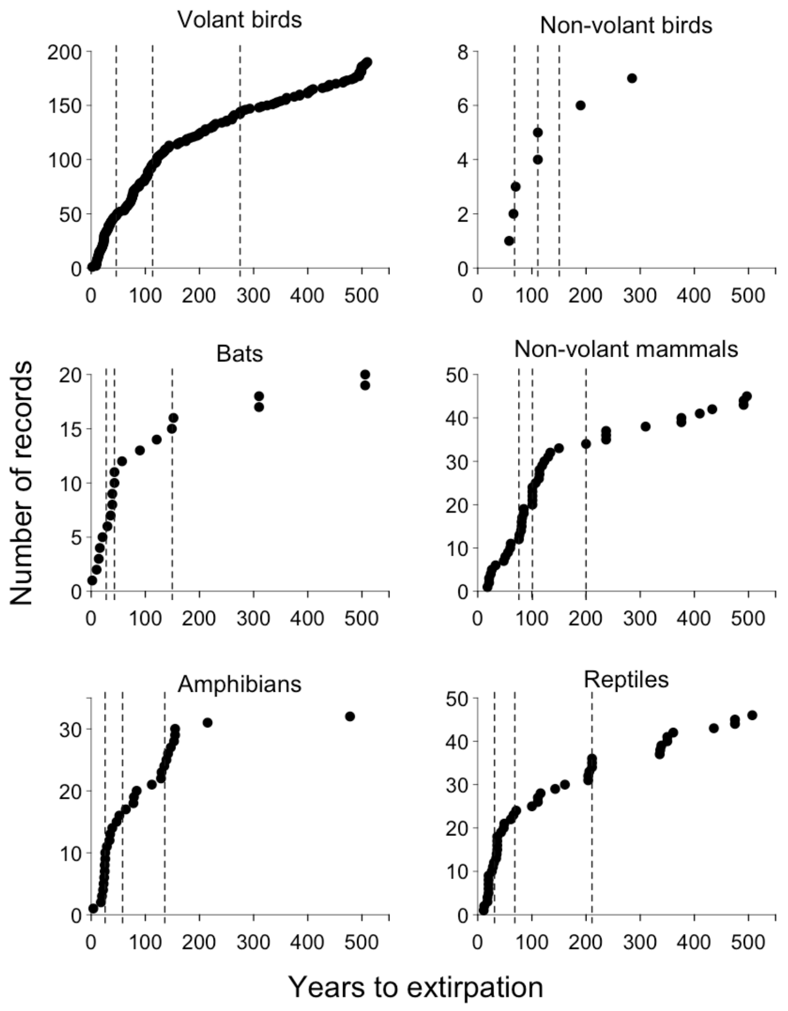

Supplementary Figure 5. Timing of population extirpations. Cumulative distribution plots show the timespan over which native vertebrate population extirpations took place, for all populations with associated information about the known or estimated year of extirpation (volant birds, n=190; non-volant birds, n=7; bats, n=20; non-volant mammals, n=45; amphibians, n=32; reptiles, n=46). A single point is shown in cases where multiple populations of a native species group were declared extirpated in the same year; this is consistent with the GEE model formulation, which grouped the data by island. Dotted lines show the 25th, 50th, and 75th percentiles for the number of years to extirpation for each native species group.

Supplementary Tables

Supplementary Table 1. Summary data for all islands in the dataset (n=1,024).

	Min.	1st Qu.	Median	Mean	3rd Qu.	Max.	NAs
Area (km^2)	0.0	0.6	9.3	4694.0	187.2	783400	0
Elevation (m)	0.3	16.8	83.7	382.5	454.2	4613.0	0
Mean annual temperature (C)	-0.9	15.9	23.8	20.4	26.0	29.1	6
Temperature seasonality	1.1	9.6	17.3	21.3	28.7	114	9
Total annual precipitation (mm)	0	702.1	1326	1459	2062	5441	9
Number of threatened species present	1	1	1	2.6	2	97	0
Number of introduced mammal types present	0	1	2.0	2.9	5	10	-

Supplementary Table 2. Classification of invasive mammal taxa.

Invasive group classification	Family & genus names	Common names
Dogs	Canis	Domestic dog
	Otocyon	Fox
	Vulpes	Wolf
Cats	Felis	Domestic & feral cat
	Caracal	Caracal
Mustelids & Mongooses	Martes	Marten
	Mustela	Weasel
	Neovison	Mink
	Atilax, Cynictis, Galerella, Herpestes	Mongoose
Pigs	Sus	Pig/boar
	Pecari	Peccary
Medium-sized omnivores	Atelerix	African hedgehogs
	Erinaceus	European hedgehog
	Viverra, Civettictis, Paradoxurus, Viverricula	Civet
	Genetta	Genet
	Dasyurus	Quoll
	Thylamys	Opossum
	Nasua	Coati
	Procyon	Raccoon
	Didelphis	American opossum
	Tenrec (family)	Tenrecs
	Trichosurus	Brushtail possum
Small omnivores	Ammospermophilus, Atlantoxerus, Spermophilus	Ground squirrel
	Tamias	Chipmunk
	Callosciurus, Funambulus, Heliosciurus, Sciurus	Squirrel
Primates	Callithrix	Marmoset
	Cercopithecus, Chlorocebus	Monkey
	Macaca	Macaque
Large herbivores	Antidorcas	Springbok
	Antilope	Blackbuck
	Raphicerus	Steenbok
	Damaliscus	Antelope
	Rangifer	Reindeer
	Boselaphus	Nilgai

	Taurotragus	Eland
	Bison	Bison
	Bos	Cattle
	Bubalus	Buffalo
	Camelus	Camel
	Capra, Oreamnos	Goat
	Hemitragus	Tahr
	Ammotragus, Ovis	Sheep
	Elaphas	Elephant
	Equus	Horse
	Lama	Llama
	Rusa, Axis, Cervus, Dama, Odocoileus	Deer
	Tayassu	Peccary
	Wallabia, Pegrogale, Macropus	Wallaby
Medium-sized herbivores	Cavia	Guinea pig
	Cuniculus	Paca
	Dasyprocta	Agouti
	Hydrochaeris	Capybara
	Phalanger	Cuscus
Lagomorphs	Lepus	Hare
	Oryctolagus, Sylvilagus	Rabbit
Rats	Rattus	Rat
	Cricetomys	
Mice	Mus, Peromyscus, Abrothrix, Apodemus	Mouse
	Eliomys	Dormouse

Supplementary Table 3. GEE model coefficients. C/V = Class/volancy (categorical variable with six levels; amphibian is the intercept). "+" refers to the presence of humans or introduced mammals.

	**Model Term**	**Coefficient**
**Main Effects**	Intercept	-5.261659601
	C/V: Bat	2.047784668
	C/V: Bird	3.385503305
	C/V: Non-volant bird	0.785890569
	C/V: Reptile	2.744908587
	C/V: Non-volant mammal	4.218883078
	Island area (log)	0.123392376
	Annual precipitation	0.000289714
	Humans+	0.548611701
	Rats+	0.741243374
	Cats+	0.530254103
	Pigs+	0.779079964
	Mustelids/mongooses+	0.449417439
**Interactions**	C/V Bat x log Area	-0.018639339
	C/V Volant bird x log Area	-0.14571061
	C/V Non-volant bird x log Area	-0.059337471
	C/V Non-volant mammal x log Area	-0.180046972
	C/V Reptile x log Area	-0.047188852
	C/V Bat x Precipitation	-0.000441254
	C/V Volant bird x Precipitation	-0.000272581
	C/V Non-volant bird x Precipitation	0.0000283
	C/V Non-volant Mammal x Precipitation	-0.00086097
	C/V Reptile x Precipitation	-0.000953778
	C/V Bat x Cat+	-0.512557599
	C/V Volant bird x Cat+	-0.351199304
	C/V Non-volant bird x Cat+	1.153571888
	C/V Non-volant mammal x Cat+	-0.093296716
	C/V Reptile x Cat+	0.11909212
	C/V Bat x Pig+	0.285790012
	C/V Volant bird x Pig+	-0.509597141
	C/V Non-volant bird x Pig+	-0.187092745
	C/V Non-volant mammal x Pig+	0.017078176
	C/V Reptile x Pig+	-0.970907894
	Rat+ x log Area	-0.075905496

Supplementary Table 4. Error statistics from K-fold (K=10) cross-validation for the final chosen model. Cross-validation was repeated 1,000 times to produce 10,000 runs from which to calculate error statistics. The minimum, mean, and maximum values for each statistic across the 10,000 runs is shown.

	Minimum	Mean	Maximum
Mean prediction error	-0.557200	0.005455	0.319500
Mean absolute prediction error	0.2810	0.3311	0.3647
Standard deviation of prediction error	0.3720	0.4299	0.6049
Mean square error	0.1401	0.1903	0.3662

Supplementary Table 5. Logistic thresholds for predicting population extirpation and persistence. Shown are the number and proportion of predicted population extirpations for the 1,998 extant populations in the data, under the minimum and maximum false-positive rates (best and worst model performance) associated with each threshold value. Also shown are the proportion of extirpations that could be prevented by invasive mammal eradication with each threshold, under the minimum and maximum false-positive rates. The values in bold (threshold = 0.21) are reported in the main text; two additional thresholds (0.15 and 0.25) were tested to determine the sensitivity of the results to threshold choice.

Threshold value	True positive rate (TPR)	False positive rate (FPR)	Maximum predicted extirpations (Min FPR)		Minimum predicted extirpations (Max FPR)		% extirpations prevented (Min FPR)	% extirpations prevented (Max FPR)
			#	%	#	%		
0.25	0.7	0.34	618	31%	408	20%	97%	47%
**0.21**	**0.8**	**0.47**	**896**	**45%**	**475**	**24%**	**75%**	**41%**
0.15	0.9	0.59	1111	56%	455	23%	56%	37%

Supplementary Methods

Native and non-native island species dataset

Island breeding records for threatened and extinct terrestrial vertebrates in the Threatened Island Biodiversity (TIB) Database were linked to geographic island locations using the Global Islands Database (GID)[1], a spatial dataset of more than 180,000 islands larger than 1 km^2. When an island's identity and/or location were unclear, we used Google Earth[2], geographic and political information found in the literature, and expert communications to obtain and validate island information. For islands smaller than 1 km^2, we used this information to manually add additional breeding islands to the spatial dataset.

We populated the TIB Database using information from peer-reviewed literature, reports, and expert communications to determine whether a species currently persists on or has been extirpated from each of its historic breeding islands (see [12] for details). We excluded cases where a species' historic or current breeding status on an island was unknown or where records were deemed unreliable. We defined extirpation to include cases in which: (1) a species has not been found during surveys on one of its historic breeding islands since 1990, or (2) species experts or the IUCN Red List account define a species as being "potentially extirpated" from an island. We classified these "likely" extirpations as extirpation rather than persistence because: (1) there is reasonable evidence that these species have been extirpated from an island, and (2) if they do persist there, the populations are presumably at such low densities that the probability of long-term persistence is low[3].

The above assumption almost certainly leads to the misclassification of some extant populations as extirpated, as it is more difficult to confirm a species' absence on an island than its presence. These misclassifications could potentially bias the results of our analysis. Therefore, we reran the analysis with all "potentially extirpated" populations classified as "present." The original dataset (as presented in the main text) contained 651 extirpations out of a total 2,656 native populations (24.5% of populations extirpated). 144 of the populations we defined as extirpated were in the "potentially extirpated" category. When we reclassified these populations as extant, the modified dataset had 507 extirpations (19% of populations extirpated). We built a model using this modified dataset and the same model selection procedure presented in the main text. The resulting model was very similar to the one presented in the main text: it included the same main effects, with the addition of average annual temperature. Most of the interactions were also the same, with the exception that the class/volancy*felid interaction term was replaced by class/volancy*mustelid/mongoose. We consider these minor changes, and based on this and the reasoning above, we conclude that our assumption is the appropriate choice for this study.

We compiled data on two biological traits of native threatened species that could influence their probability of extinction in general and their vulnerability to introduced mammals in particular: flight ability and body mass. All bats were classified as volant, and all other mammals as non-volant. We classified birds as volant or non-volant using BirdLife International's species factsheets[4]. Many other intrinsic traits of native species, such as home range size, ground vs. arboreal nesting, and migration patterns likely also influence the vulnerability of insular species to invasive mammals, but a lack of sufficient data on these variables for all species in our data precluded us from investigating them. We deemed body mass and flight ability to be

fundamentally important intrinsic factors because numerous studies have shown them to be primary determinants of species extinction risk on islands[5–9]. To ensure that the inclusion of volancy in addition to taxonomic class did not greatly affect our results, we also ran the analysis with just the four native taxonomic groups (amphibians, reptiles, birds, and mammals). The resulting model was nearly identical to the model we present in the main text, except that it lacked two interaction terms: class/volancy*area and class/volancy*pig. This suggests that the effects of island area and invasive pigs on insular birds and mammals are mediated by native species' flight ability. Disentangling the effects of area and invasive pigs on volant vs. non-volant species is an important component of (1) understanding historic and current extinction patterns and (2) more accurately predicting the outcome of management actions for each native group. Therefore, in the main text we present only the model that incorporates flight ability.

Body mass data were gathered from scientific literature and online resources (Supplementary Data 2). For birds and mammals, where minimum and maximum mass were given or male and female values were listed separately, we used the arithmetic mean of the two values. For amphibians and reptiles, the standard metric for body size is snout-vent length (SVL), and body mass data were not available for most species. Thus, for species lacking information on body mass, we used SVL as well as total animal length (TL) when this was provided. We used allometric equations developed for each taxon to convert the minimum and maximum SVL or TL to body mass estimates[10], and then took the arithmetic mean of the minimum and maximum mass estimates to obtain a single body mass estimate for each species. When information on the body mass (mammals and birds) or SVL (amphibians and reptiles) of a particular species was unavailable, we used either: (1) the body mass or SVL of a similarly sized congener, when such

information was available, or (2) the mean value of the body masses or SVLs of many similarly sized congeners.

Body mass data were unavailable for some native species that are extinct or are very rare and have never been weighed. We excluded these species from the analysis because the model selection procedure can only accommodate complete rows of data. Of a total 1,257 native threatened or extinct species in the original dataset, mass data were unavailable for 53 species (4%). Despite this being a relatively small proportion of the data, it is possible that a taxonomic bias in the omitted species could influence the results. In particular, most of the species for which mass data were unavailable were extinct birds and mammals. Only two amphibian and two reptile species lacked mass data, because for these taxa we calculated body mass from animal length, which can be obtained from preserved specimens and are thus available even for most extinct species. We ran the full model selection procedure on the subset of data for which body mass data were available, and threatened species body mass was not selected in any of the top models, indicating that omitting it from the analysis did not affect the results.

We recorded the presence or absence of all non-native mammals on islands for which we could find information[11,12]. We identified ~160 invasive mammals that had become established on islands and classified them into 12 groups based on a combination of feeding guild, taxonomy, body size, and other traits (Table 1 and Supplementary Table 2). Our goal was to achieve a balance between creating as few groups as possible to maximize the representation of each group across islands (i.e. sample size within strata) and as many groups as needed to distinguish between impacts of different mammal types. For example, we grouped primates separately from

other omnivores such as rodents, raccoons, and possums due to their primarily arboreal nature and unique behavior. We divided carnivores into three groups—felids, canids, and mustelids/mongooses—because we predicted they would have different impacts on native species based on life history, behavior (e.g. solitary or pack hunters), and home range size. Although mongooses are most closely related to civets and genets (family Herpestidae), which we grouped with other medium-sized omnivores based on diet, we grouped mongooses with mustelids because the common island invasive species in both these groups are largely or entirely carnivorous. We separated rodents from the other omnivore groups due to their generally smaller body sizes, their high densities and population growth rates on islands, and their broad omnivorous diets. In addition, rodents are the most common target of invasive mammal eradication efforts[13], so their unique impacts are particularly important to understand from a conservation perspective. We classified rats and mice separately to tease apart potentially different impacts of these groups due to differences in body size and behavior, and because rats are targeted for eradication more frequently than mice. The two most ubiquitous rat species on islands, *Rattus rattus* and *R. norvegicus*, differ in climbing ability (strong and weak climbers, respectively), and may therefore affect arboreal and terrestrial island species differently. However, the particular species of rat present on many of the islands in our dataset is unknown, making it impossible to distinguish their individual impacts.

Island attributes dataset

For most islands, we obtained data on island climate and elevation from the publically available BioClim Dataset (resolution 30 seconds)[14]. We obtained a single estimate for each climatic variable per island by calculating the arithmetic mean of the center points of all raster grid cells

located within the boundary of an island polygon. A disadvantage of this approach is that more climatic information is lost for larger islands than for small ones, particularly for islands that span large latitudinal gradients, are topographically complex, or have distinct wet and dry regions. However, our distribution data for native and introduced species were at the scale of whole islands, thus the island was the geographic unit of our analysis and we used island-level values for each geographic and climatic variable.

Some islands in the TIB Database had no overlapping raster grid cells in the BioClim climate or elevation datasets. For islands located less than 100 km from the nearest continent or island, we used climate values from the nearest grid cell in the BioClim raster. There were no islands with climate data within 100 km of the Bounty Islands (New Zealand), Pitcairn and Henderson Islands (UK Overseas Territories), or the islands of French Polynesia, so we obtained climate data for these groups from outside data sources[15–17]. For most islands lacking GIS elevation data, we obtained maximum elevation values from the literature[18–24] and from Google Earth[2]. We were unable to find elevation data for some small islands using any of these sources, and we estimated the maximum elevations of these islands using linear interpolation. Specifically, for each island lacking elevation data, we identified all the islands in our dataset that are located in the same archipelago and share the same geologic history (and therefore likely have similar topographic characteristics) as that island. We used the area-elevation relationship of the other islands in the archipelago to interpolate the island's maximum elevation based on its area.

There were nine islands in the dataset for which we were unable to find or reasonably estimate temperature, precipitation, or temperature seasonality. We excluded these islands because the

model selection procedure can only analyze complete rows of data. However, these omitted islands represent a very small proportion of the total dataset and therefore are unlikely to influence the results of our analysis. Each of these nine islands contained one threatened vertebrate species; thus, we excluded nine native populations on nine islands, out of a total 2,657 native populations on 1,024 islands in the full dataset. The small proportions of omitted data due to missing climate information (<0.5% of native populations and <1% of islands) is unlikely to influence the results of the analysis beyond having minute effects on model coefficients.

Island isolation is a primary determinant of species immigration rates and equilibrium species richness[25]. However, we did not include isolation as a covariate in our model for several reasons. First, island biogeography theory makes no predictions about the effect of isolation on short-term extinction risk, but rather focuses on long-term equilibrium species richness due to intrinsic extinction and immigration rates. Second, the only other global study that measured correlates of extinction risk specifically for threatened insular species found no relationship between isolation and either endemism rates or the severity of impacts from invasive mammals[26]. Finally, island isolation is unlikely to be related to invasion pressure because most mammal introductions resulted from intentional or unintentional human actions. Rats and mice, for example, are common stowaways on ships, and were introduced to extremely remote islands via landings and shipwrecks throughout the exploration and whaling periods of the last 500 years and earlier[27,28]. Therefore, we did not consider isolation as a driver of short-term population extinction risk for the species and islands in our dataset.

The ecology of some very large islands in our dataset may, in some ways, resemble that of

continents more than that of small islands; if this is true, including these islands in our analysis could obscure patterns that are unique to smaller islands. We kept even the largest islands (e.g. New Guinea, Borneo, Madagascar) in our analysis for three main reasons. First, like many smaller islands, even these very large islands contain unique radiations of taxa that evolved in conditions different from those found on continents, often including a lack or paucity of native mammals or terrestrial predators. Thus, we hypothesized that native species on large islands would show similar patterns of extirpation and persistence, with respect to our model covariates, to those on smaller islands. Second, the only other global analysis of threats to insular species from invasive mammals also included many of the world's largest islands[26]. Third, our analysis of model residuals and outliers identified islands with the strongest influence on model results and predictions. The only islands identified as outliers were Sri Lanka (the 16th largest island in the data) and Maria Madre (a relatively small island [152 km^2] located off the Pacific coast of Mexico); none of the very large islands in the data were identified as outliers.

Nevertheless, to ensure that including the large islands did not strongly influence our results, we ran the analysis with the nine largest islands in the dataset excluded (New Zealand's North and South Islands, Java, Sulawesi, Honshu, Sumatra, Madagascar, Borneo, and New Guinea). We used the same model selection procedure as described in the main text, and the resulting model contained 11 main effects and 9 interaction terms. In addition to the main effects and interaction terms present in the final model presented in the main text, this model contained three additional main effects (area-elevation residuals, introduced canids, and temperature) and four interaction terms: temperature*mustelid/mongoose, class/volancy*mustelid/mongoose, class/volancy*canid, class/volancy*temperature. We speculate that the inclusion of more terms and interactions in this

model is due to a smaller effect of island area, which allows some of the other variables and interactions to have greater importance in the model. However, interpreting a model of this complexity is not feasible because the individual and combined roles of each term and interaction cannot be untangled. An analysis focusing on a smaller subset of islands – based on size or any other geographic characteristic – would be an interesting follow-up to our study, to better understand the nuances of different variables' impacts and interactions at smaller spatial scales. For this global-scale study, however, we considered the very large islands to be important components of our analysis and results.

Extirpation patterns may differ across archipelagos, island regions, or ocean basins due to intrinsic factors (e.g. species assemblages in a region may share a more recent taxonomic history and may therefore be similarly vulnerable to anthropogenic threats) and/or external factors (e.g. different history of colonization by humans or invasive mammals). However, we did not include geographic region as a factor in our model because it is not directly relevant to the questions our study was intended to address. We chose a set of abiotic predictor variables and interactions for which we had specific hypotheses about their effects on extirpation patterns (Tables 1 and 2), and geographic region would not provide further ecological or mechanistic insights into the factors driving extirpations. For example, our hypothesis that insular non-volant birds are vulnerable to predation by invasive rats or cats, or that native omnivores would be negatively affected by competition with invasive omnivores, applies to islands in all regions of the globe. These impacts may be mediated by island topography or climate, which we included in the analysis with relevant hypotheses, but are not likely mediated by geographic region. In addition, defining boundaries between archipelagos or island regions would be tricky and could be prone

to bias or arbitrary delineation of regions. For example, the islands of the Pacific Ocean could be divided into any number of groups based on distance to the nearest continent, geologic or biological affinity with other islands or archipelagos, latitude or longitude, or larger-scale ocean basin. Thus, any choice of island regions could bias the results of our analysis and would be unlikely to advance our goal of understanding extirpation patterns and invasive mammal impacts at a global scale.

The TIB Database includes information on human habitation for most islands. Precise population data are not available for many islands, so each island is classified into one of six human population categories using whatever information is available. Islands are classified as uninhabited or as having permanent human populations numbering 1-10, 11-100, 101-1,000, 1001-10,000, or >10,000. For many islands, we were unable to find data specific enough to classify them into any of these categories. However, in all cases we were able to asses whether humans likely maintain permanent settlements on islands by conducting Web searches (e.g. many islands are tourist destinations with hotels and other infrastructure) and searching Google Earth images for buildings, roads, agriculture, or other signs of human habitation. Based on this information, we supplemented the information obtained from the TIB Database and classified all islands in the dataset as either inhabited or uninhabited.

Extirpation probability analysis

We constructed logistic models to investigate global patterns of population extirpation and persistence for threatened island vertebrates. The binary response variable was the extirpation or persistence of a native species on one island. Many islands contain multiple native threatened

species, each with its own record of persistence or extirpation, while others have only one threatened species. Similarly, some native species occur (or occurred historically) on many islands, while others are (or were) single-island endemics.

We were unable to document the timing of extirpation events with enough precision to include time as a factor in our analysis, or to use our model to predict the timing of future extirpations. About half the extirpation records in our data had associated timing information, which typically consisted of the last year a species was documented on an island and the year the species was declared or presumed extirpated. For populations with sufficient data, we calculated the timespan over which extirpations occurred (i.e. the number of years between these two events), which ranged from <5 years to ~500 years (all species included in the IUCN Red List persisted until after 1500 AD, thus the maximum time to extirpation was 510 years) (Supplementary Fig. 5). This wide range of values, along with the lack of temporal data for half of the extirpations and the geographic and taxonomic bias inherent in historic survey data, prevented us from incorporating time in the analysis.

Supplementary References

1. UNEP-WCMC. Global Islands Database. (2013).
2. Google. Google Earth. (2011). at <www.google.com/earth>
3. Millenium Ecosystem Assessment. *Ecosystems and Human Well-being: Biodiversity Synthesis*. (2005).
4. BirdLife International. Species Factsheets. at <http://www.birdlife.org/datazone/home>
5. Holdaway, R. N. New Zealand's pre-human avifauna and its vulnerability. *N. Z. J. Ecol.* **12,** 11–25 (1989).
6. Alcover, J. A., Sans, A. & Palmer, M. The extent of extinctions of mammals on islands. *J. Biogeogr.* **25,** 913–918 (1998).
7. Steadman, D. W. Prehistoric extinctions of Pacific Island birds: biodiversity meets zooarchaeology. *Science* **267,** 1123–1131 (1995).
8. Boyer, A. G. Consistent ecological selectivity through time in Pacific Island avian extinctions. *Conserv. Biol.* **24,** 511–519 (2010).
9. Whittaker, R. J. & Fernandez-Palacios, J. M. *Island biogeography: Ecology, evolution, and conservation*. (Oxford University Press, 2007).
10. Pough, F. H. The advantages of ectothermy for tetrapods. *Am. Nat.* **115,** 92–112 (1980).
11. Threatened Island Biodiversity Database Partners. The Threatened Island Biodiversity Database: developed by Island Conservation, Univ. of California Santa Cruz Coastal Conservation Action Lab, BirdLife International and IUCN Invasive Species Specialist Group. Version 2012.1. (2012). at <http://tib.islandconservation.org>
12. Spatz, D. R. *et al.* The biogeography of globally threatened seabirds and island conservation opportunities. *Conserv. Biol.* **28,** 1282–1290 (2014).
13. Keitt, B. S. *et al.* in *Island invasives: eradication and management* (eds. Veitch, C. R., Clout, M. N. & Towns, D. R.) 74–77 (IUCN, Gland, Switzerland, 2011).
14. Hijmans, R. J., Cameron, S. E., Parra, J. L., Jarvis, P. G. & Jones, A. Very high resolution interpolated climate surfaces for global land areas. *Int. J. Climatol.* **25,** 1965–1978 (2005).
15. The World Bank. The Climate Change Knowledge Portal. (2013). at <http://sdwebx.worldbank.org/climateportal/index.cfm>
16. Vacations to Go: Bounty Islands, New Zealand. (2013). at

<http://www.vacationstogo.com/cruise_port/Bounty_Islands__New_Zealand.cfm>

17. Brooke, M. de L., Hepburn, I. & Trevelyan, R. J. Henderson Island World Heritage Site: Management Plan 2004-2009. (2004).
18. Arata, J., Robertson, G., Valencia, J., Xavier, J. C. & Moreno, C. A. Diet of grey-headed albatrosses at the Diego Ramirez Islands, Chile: ecological implications. *Antarct. Sci.* **16,** 263–275 (2004).
19. Kagiampaki, A., Triantis, K., Vardinoyannis, K. & Mylonas, M. Factors affecting plant species richness and endemism in the South Aegean (Greece). *J. Biol. Res.* **16,** 282–295 (supp) (2011).
20. Kirkwood, R. *et al.* Estimates of Southern Rockhopper and Macaroni Penguin numbers at the Ildefonso and Diego Ramírez Archipelagos, Chile, using quadrat and distance-sampling techniques. *Waterbirds* **30,** 259–267 (2007).
21. Melnick, D., Bookhagen, B., Echtler, H. P. & Strecker, M. R. Coastal deformation and great subduction earthquakes, Isla Santa María, Chile (37°S). *Geol. Soc. Am. Bull.* **118,** 1463–1480 (2006).
22. Morrison, R. J. & Woodroffe, C. D. The Soils of Kiritimati (Christmas) Island, Kiribati, Central Pacific: New information and comparison with previous studies. *Pacific Sci.* **63,** 397–411 (2009).
23. Schlatter, R. in *A directory of wetlands in Oceania* (ed. Scott, D. A.) (International Waterfowl and Wetlands Research Bureau and Asian Wetland Bureau, 1993).
24. Office of Te Beretitenti. *21. Tabuaeran*. (2012).
25. MacArthur, R. H. & Wilson, E. O. *The equilibrium theory of island biogeography*. (Princeton University Press, 1967).
26. Walsh, J. C. *et al.* Exotic species richness and native species endemism increase the impact of exotic species on islands. *Glob. Ecol. Biogeogr.* **21,** 841–850 (2012).
27. Long, J. L. *Introduced mammals of the world*. (CABI, 2003).
28. Atkinson, I. A. E. in *Conservation of island birds* (ed. Moors, P. J.) 35–81 (International Council for Bird Preservation, Technical Publication No. 3, 1985).
